# Histoplasmosis with mediastinal lymphadenopathy in an immunocompetent municipal water department employee in Texas: a case report

**DOI:** 10.3389/fcimb.2026.1808506

**Published:** 2026-05-12

**Authors:** Tianci Wang, Austin Fajkus, Morgan Fairweather

**Affiliations:** 1 Anne Burnett Marion School of Medicine, Texas Christian University, Fort Worth, TX, United States; 2 Department of Internal Medicine, Texas Health Harris Methodist Hospital Fort Worth, Fort Worth, TX, United States

**Keywords:** disseminated histoplasmosis, endemic mycosis, *Histoplasma capsulatum*, occupational water exposure, *Rickettsia typhi*

## Abstract

**Background:**

Histoplasmosis is an endemic mycosis that primarily affects the lungs, commonly found in the central and eastern United States, particularly the Ohio and Mississippi River valleys. While disseminated histoplasmosis (DH) is typically associated with immunocompromised individuals, rare cases have been reported in immunocompetent hosts following high-risk environmental exposures, particularly in high-risk occupational settings.

**Case presentation:**

We report a case of a 22-year-old immunocompetent male employee of a municipal water department in Texas who presented with fever, pleuritic chest pain, and progressive dyspnea. Initial workup suggested pneumonia, but lack of response to empiric antibiotics and evolving bilateral pulmonary infiltrates prompted further evaluation. A detailed exposure history revealed recent occupational exposure involving disruption of soil and debris in an area with stagnant water in an abandoned warehouse. Subsequent testing confirmed the diagnosis of histoplasmosis with positive urine antigen, elevated serum *Histoplasma* titers, and lymph node biopsy demonstrating granulomatous inflammation with yeast forms consistent with *Histoplasma* outside the lung parenchyma, raising concerns for disseminated disease. This patient was also incidentally found to have positive serum *Rickettsia typhi* antibodies. He was treated with oral itraconazole and demonstrated clinical improvement.

**Conclusion:**

Both pulmonary and disseminated histoplasmosis should be included in the differential diagnosis for febrile respiratory illness in individuals with high-risk occupations regardless of immune status, including in regions not classically considered endemic. Early recognition and antifungal therapy are essential to prevent complications and ensure recovery.

## Introduction

Histoplasmosis is an endemic mycosis caused by *Histoplasma capsulatum* (including var. *capsulatum* and var. *duboisii*), a dimorphic fungus that thrives in damp soil enriched with bat or bird droppings, and infection typically occurs through inhalation of aerosolized microconidia (Histoplasmosis - clinicalKey; Wheat et al., 2007). Although historically considered endemic to the Ohio and Mississippi River Valleys based on histoplasmin skin testing data from the 1950s (Develoux et al., 2021), histoplasmosis has now been reported across increasingly wide geographic areas, including the central and eastern United States, as well as parts of Central and South America, Africa, and South Asia (CDC, 2024a). In the United States, histoplasmosis has an estimated incidence of 1 to 7 cases per 100,000 population based on the most recent surveillance data from the U.S. Centers for Disease Control and Prevention, and mortality rates vary widely depending on disease severity and host immune status (Develoux et al., 2021; CDC, 2024a; CDC, 2024b; Dao et al., 2024).

While most infections are asymptomatic, symptomatic cases often present with flu-like illness characterized by fever, malaise, dry cough and chest discomfort; recovery is typically expected within 30 days (Barros et al., 2023). Severe disease is more likely to occur with high inoculum exposure or impaired host immunity and may progress to disseminated histoplasmosis, defined by the Infectious Diseases Society of America (IDSA) as having physical, radiographic findings or laboratory evidence of extrapulmonary involvement (Histoplasmosis - clinicalKey; Pappas et al., 2025). Radiographic imaging typically reveals diffuse reticulonodular infiltrates and hilar lymphadenopathy, while diagnosis is supported by antigen testing, serology, fungal culture, or tissue histopathology (Thieme E-journals - seminars in respiratory and critical care medicine / full text). Timely recognition and antifungal initiation are essential to prevent morbidity.

Although disseminated histoplasmosis (DH) is classically associated with immunosuppressed patients, cases have been documented in otherwise healthy adults following high-inoculum exposure events, including construction, soil disruption, or water infrastructure work (de Perio et al., 2021). In this report, we present a rare case of acute pulmonary histoplasmosis with possible disseminated disease in an immunocompetent 22-year-old municipal water department worker with no known relevant past medical history, who was also found to have positive serologic titers for typhus fever.

## Case presentation

A 22-year-old male with a history of benign essential hypertension presented to the emergency department (ED) with four days of fever up to 102.6°F, dyspnea on exertion, pleuritic chest pain, dizziness, malaise, and nausea. He denied cough and upper respiratory symptoms, sick contacts, recent travel, pets or exposure to exotic animals. His social history was notable for occupational exposure to water infrastructure environments through his work at the local water department.

**Figure 1:**
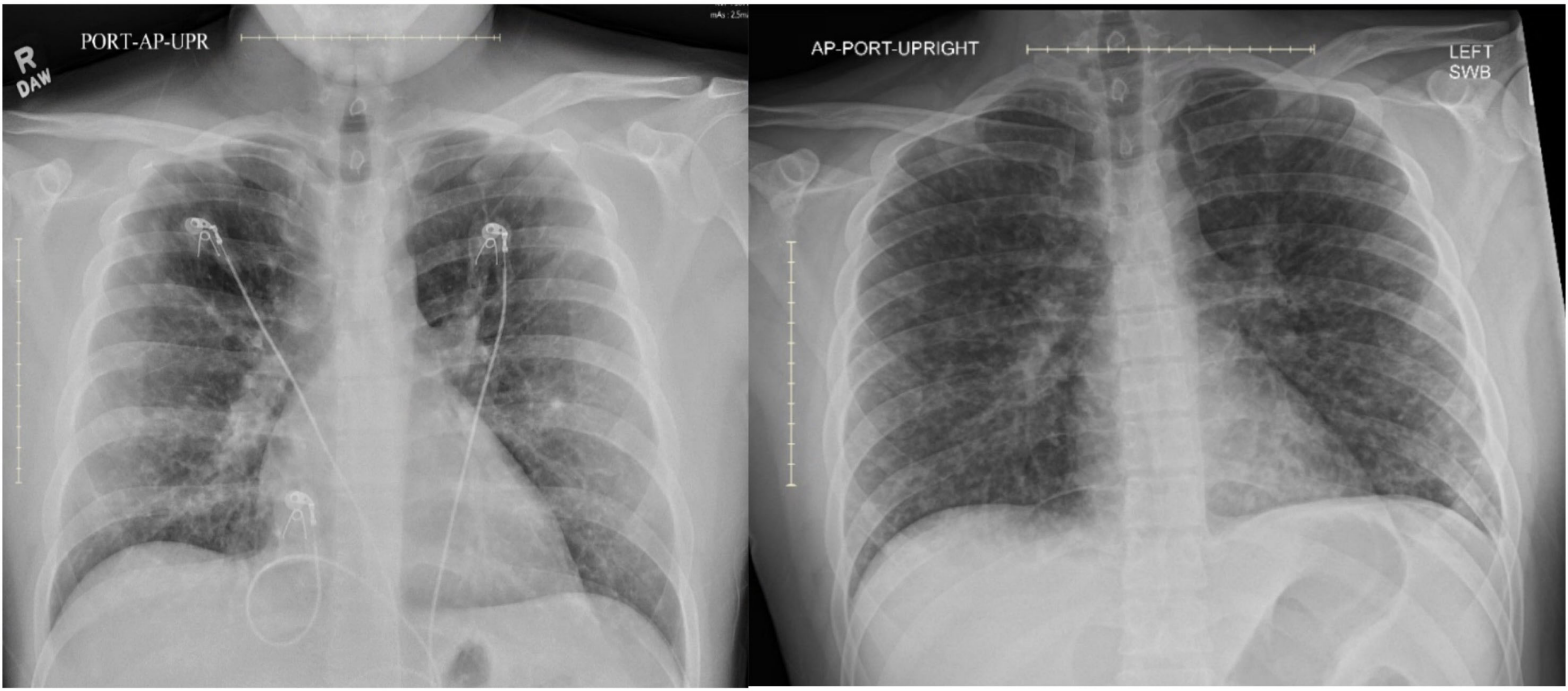
Patient’s chest radiography findings on admission (left) and several days after (right), revealing progressive diffuse reticulonodular opacities.

In the ED, initial examination was notable for hypertension (171/115 mmHg), tachycardia (155 bpm), and fever (102.6°F). The remainder of the exam was unremarkable, with normal breath sounds and oxygen saturation of 96% on room air, no reproducible chest wall pain, and no lymphadenopathy. Initial laboratory evaluation revealed a normal white blood cell count (10,000/μL), elevated procalcitonin (0.56 ng/mL) and D-dimer (0.9 µg/mL). A comprehensive metabolic panel was within normal limits. Testing for COVID-19, influenza, and an extended respiratory viral panel was negative. Chest radiography revealed reticulonodular opacities scattered throughout the right lung (**Figure 1**, left panel). Respiratory and blood culture samples were obtained.

Given his pleuritic chest pain and tachycardia, an electrocardiogram was obtained and showed sinus tachycardia at 134 bpm without ST-T abnormalities, and three serial high sensitivity troponins were <5. Computed tomography angiography (CTA) of the chest was performed to evaluate for pulmonary embolism due to findings of tachycardia, pleuritic chest pain, elevated D-dimer, and a revised Geneva Score of 5 points (heart rate >95 bpm) corresponding to a moderate-risk category. The CTA did not identify pulmonary embolism but did demonstrate diffuse peribronchovascular and subpleural nodular opacities with mediastinal and hilar lymphadenopathy, raising concerns for sarcoidosis. The patient was initiated on empiric ceftriaxone and azithromycin and was admitted for management of presumed bacterial pneumonia. Pulmonology was consulted to consider bronchoscopy in light of the abnormal CTA findings.

After one day of antibiotic therapy, the patient remained febrile and tachycardic and developed a cough with intermittent hypoxia requiring 2L of oxygen via nasal cannula. Repeat blood testing revealed new hypoalbuminemia (nadir 2.9 g/dL) and mild transaminitis: ALP 160 U/L, AST 99 U/L, ALT 74 U/L, with normal bilirubin (0.4 mg/dL). Repeat chest radiography showed progression of reticulonodular infiltrates (**Figure 1**, right panel). Pulmonology specialists considered sarcoidosis as unlikely to explain the patient’s acute presentation, and recommended expanding the differential diagnosis to include atypical infections. Urine Legionella and pneumococcal antigens were obtained and were negative.

Infectious disease consultation elicited additional occupational history. The patient reported that in the course of his regular duties as a municipal water department employee, he visited a derelict warehouse about 5 days prior to symptom onset. He reported the site had several areas of standing water, and his work involved disturbing the soil and debris at the worksite. He denied known exposure to rodents, bats, or insects. A broad infectious workup was then pursued, including viral infections, endemic mycoses, cryptococcosis, and zoonotic infections. Antimicrobial therapy was broadened to intravenous cefepime 1 g every 6 hours and oral doxycycline 100 mg twice daily.

The complete results of the infectious workup are presented in **Table 1**. Notable findings included a positive urine *Histoplasma* antigen and elevated *Histoplasma* yeast antibody titer (1:32). Serologic testing for *Rickettsia typhi* also showed positive IgM and IgG titers of 1:256, although pertinent clinical signs and symptoms such as petechial rash, abdominal pain, thrombocytopenia or elevated blood urea nitrogen levels were not observed. Blood and sputum cultures both returned negative. Given the serologic findings and ongoing diagnostic uncertainty, the patient was initiated on empiric itraconazole and doxycycline to provide coverage for possible histoplasmosis and rickettsial infection.

**Table 1:** Summary of infectious disease workup.

Test	Result	Test	Result
(1,3)-beta-D-glucan	<31	Mycoplasma Pneumoniae PCR	Negative
Aspergillus Antibody	<1.8	Q-Fever Antibody Phase I, IgG	Negative
Blastomyces Antibody	<0.4	Q-Fever Antibody Phase II. IgG	Negative
Bordetella Pertussis, by PCR	Negative	Aspergillus Galactomannan Ag	0.000
Coccidioides Antibody	40.2	Aspergillus Galactomannan Index	0.00
Cryptococcal Antigen	Negative	Tularemia Antibody IgG	<1:16
Histoplasma Antigen, Urine	**Detected**	Tularemia Antibody IgM	Negative
Histoplasma Ag EIA, Urine	7.792	Typhus Fever Antibody, IgG	**1:256**
Histoplasma Mycelia Antibody	<1:8	Typhus Fever Antibody, IgM	**1:256**
Histoplasma Yeast Antibody	**1:32**	MASA by PCR	Negative
HIV 1/2 Ab + HIV 1 Ag	Non-reactive	TB GOLD	Negative
Chlamydia pneumoniae PCR	Negative		

Positive results relevant to the final diagnosis are shown in bold.

To further establish the diagnosis of histoplasmosis and assess for disseminated disease in the setting of lymphadenopathy seen on CTA, the patient underwent bronchoscopy with bronchoalveolar lavage (BAL) of the right upper lobe (RUL) and endobronchial ultrasound (EBUS)-guided transbronchial needle aspiration (TBNA) of mediastinal lymph nodes 7, 11R and 4R. Among these samples obtained, the station 7 lymph node FNA and cell block demonstrated noncaseating granulomas with yeast forms on Gomori methenamine silver (GMS) stain, consistent with histoplasmosis. Other lymph node samples were negative for malignancy or infection, and BAL specimens were negative for infection on staining and fungal culture.

Of note, *Coccidioides* antibody testing also returned elevated on initial screening. In the context of the case, the results were interpreted as cross reactivity among endemic mycoses, especially given the combination of positive urine *Histoplasma* antigen testing, yeast-phase antibodies, and lymph node histopathology, all supporting the primary diagnosis of histoplasmosis. Notably, the patient did not have any evidence of being immunocompromised on evaluation, with a normal white blood cell count, negative HIV antigen/antibody screening, and negative QuantiFERON-TB results, and denied taking any immunomodulators. More extensive testing including immunoglobulins was not conducted given low clinical suspicion.

Over the next five days, the patient demonstrated gradual clinical improvement and was discharged home to complete the course of itraconazole and doxycycline. Two weeks post-discharge, the patient reported continued need for 2–3 L of supplemental oxygen and continued to experience significant fatigue. At a subsequent follow-up appointment with his primary care physician, he reported completion of both itraconazole and doxycycline courses, with marked improvement in symptoms and a return to normal daily activities.

## Discussion

**Figure 2:**
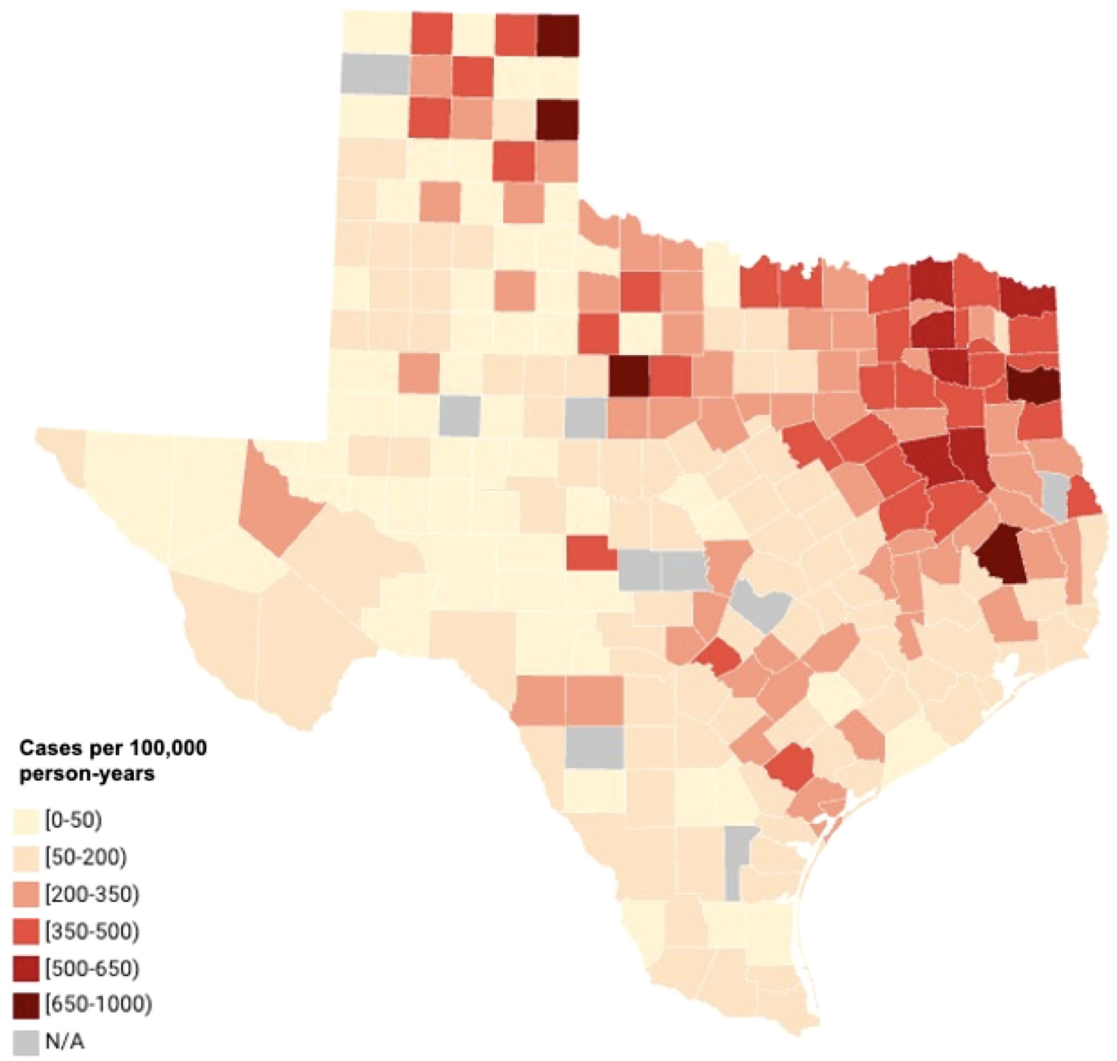
Incidence of histoplasmosis across Texas from 2007 to 2016, adapted from medicare fee-for-service beneficiary data reported by (Mazi et al., 2022). Counties are color-coded by cases per 100,000 person-years. Figure rendered with datawrapper.

Despite being considered endemic, histoplasmosis has been increasingly reported outside its traditional endemic areas due to factors such as increased travel, agricultural development, expanded use of immunosuppressive therapies, and climate change (Ashraf et al., 2020). The present case was reported in northern Texas situated in the trinity river basin. Texas is not classically emphasized in historic endemic maps, with a recent retrospective study by Benedict and Mody reporting only 4 outbreaks and approximately 50–143 cases reported between 1938 and 2013 (Benedict and Mody, 2016). However, these figures likely substantially underestimate the true prevalence, as histoplasmosis is not a reportable disease in Texas and most U.S. states (Smith, 2022). A recent analysis of Medicare fee-for-service beneficiaries by Mazi et al. identified a total of 79,749 cases of histoplasmosis in the United States between 2007 to 2016, with cases reported in 94% of U.S. states, demonstrating that histoplasmosis occurs far beyond its traditional endemic boundaries (Mazi et al., 2022). Their data revealed that more than half of counties in Texas had a histoplasmosis incidence exceeding 200 cases per 100,000 person-years, with the highest incidence observed in the northeastern region of the state, overlapping with the (Mazi et al., 2022). **Figure 2** illustrates the distribution of histoplasmosis cases across the state, adapted from dataset published by Mazi et al. Notably, regions with higher histoplasmosis prevalence appear to generally overlap with areas of dense river basins within Texas (Hydrography data and map-based data viewers), a pattern likely explained by population density as well as environmental conditions favorable for *Histoplasma* growth. Additionally, genomic epidemiological studies have further revealed that two distinct *Histoplasma* species (*H. mississippiense and H. ohiense*) circulate in the United States, with *H. mississippiense* prevalent in central and western states including Texas. Updated understanding of its distribution is crucial in ensuring the timely recognition and management of histoplasmosis, especially for cases outside its endemic areas (Tenório et al., 2024).

In this case, the patient’s occupation with the municipal water department and his recent assignment to a derelict warehouse were plausible source of exposure to *Histoplasma*. Previous literature has identified sanitation workers, construction crews, and tunnel workers as at-risk populations due to frequent soil and water disruption and exposure to spaces harboring bird or bat droppings (de Perio et al., 2021; Pappas et al., 2025). Work-related exposure is one of the most common sources of histoplasmosis in otherwise healthy adults. Between 1938 and 2013, 35 work-related histoplasmosis outbreaks were reported in the United States, accounting for approximately 33% of all documented outbreaks (Benedict and Mody, 2016). To mitigate occupational risk, the National Institute for Occupational Safety and Health (NIOSH) recommends a “hierarchy of controls” strategy to reduce work related histoplasmosis. This includes elimination of bats or birds from buildings, controlling dust generation and aerosolized dust, appropriate use of personal protective equipment (PPE), and hazard awareness and communication training for employees (de Perio et al., 2021; CDC, 2024c).

This patient’s exposure history, along with fever, dyspnea, pleuritic chest pain, pulmonary reticulonodular infiltrates and mediastinal lymphadenopathy necessitated the consideration of a wide range of differential diagnoses. Infectious etiologies considered included viral or atypical bacterial pneumonia, mycobacterial infection such as pulmonary tuberculosis, septic pulmonary emboli, fungal infections such as endemic mycoses (blastomycosis, histoplasmosis and coccidiomycosis) and cryptococcosis, as well as zoonotic infection such as tularemia, Q fever, and murine typhus. Non-infectious causes were also considered including hypersensitive pneumonitis, malignancy (e.g. lymphoma or lung cancer), sarcoidosis, and amyloidosis. Lastly, additional important non-pulmonary diagnoses including acute pulmonary embolism, pericarditis and myocardial infarction, were considered and ruled out early in his hospital course. Given the nonspecific presenting symptoms and broad possible differentials, histoplasmosis is frequently initially misdiagnosed in non-endemic or low-suspicion settings, resulting in diagnostic delays (Ubesie et al., 2013; Rog et al., 2016; Zhu et al., 2016; Specjalski et al., 2019; Benedict et al., 2020b; Benedict et al., 2020a; Miller et al., 2022). A recent study interviewing 301 patients with confirmed histoplasmosis found that 60% had seen a healthcare provider more than three times prior to receiving the correct diagnosis, and over half have received antibiotics (Benedict et al., 2020b). Given its potential to cause serious illness, the CDC recommends that clinicians consider endemic mycoses in patients presenting with symptoms of community-acquired pneumonia, especially those with risk factors such as travel or occupational exposure to environments conducive to *Histoplasma* growth (Smith, 2022).

Based on the IDSA 2025 updated guidelines, this case is best categorized as moderate acute pulmonary histoplasmosis, with concern for dissemination due to mediastinal lymph node histopathology demonstrating granulomas with yeast forms outside the primary pulmonary parenchyma (Pappas et al., 2025). Disseminated histoplasmosis is defined as involvement of extrapulmonary sites, particularly the reticuloendothelial system (e.g., lymph nodes, liver, spleen, or bone marrow), via hematogenous spread (Eichenberger et al., 2025). The patient demonstrated abnormalities in other organ systems, including elevated liver enzymes and hypoalbuminemia, although confirmatory evaluation for disseminated disease (e.g., abdominal imaging or bone marrow examination) was not pursued, as the patient had no features suggestive of moderately severe or severe disseminated disease which would alter clinical management, and showed a favorable response to itraconazole therapy. The patient was managed as having moderate acute pulmonary histoplasmosis with possible mild-to-moderate disseminated involvement. Per IDSA guidelines, both entities can be treated with itraconazole alone (200 mg 3 times daily for 3 days, then twice daily), rather than the traditional regimen for disseminated disease, which consists of liposomal amphotericin B followed by itraconazole (Wheat et al., 2007; Pappas et al., 2025).

Disseminated histoplasmosis is typically associated with immunosuppressed populations, including patients with HIV/AIDS, solid organ transplants, or prolonged corticosteroid use (Histoplasmosis - clinicalKey). Disseminated histoplasmosis in immunocompetent individuals residing outside endemic areas is rare and have only been reported a few times in the literature, typically associated with high-inoculum exposures, though some cases had no identifiable exposures (Ubesie et al., 2013; Rog et al., 2016; Ling et al., 2018; Samaddar et al., 2019; Crawford et al., 2024). Our patient was considered immunocompetent based on an overall healthy baseline and the absence of clinical or laboratory evidence of immunocompromise, including negative tuberculosis testing, negative HIV status, and no history of immunomodulator use or organ transplant. Further immunologic evaluation, such as lymphocyte subset analysis, was not performed due to low clinical suspicion for primary immunodeficiency, and demonstrated an appropriate clinical response to antifungal therapy.

Lastly, it is interesting to note that our patient had positive serologic testing (IgM and IgG) for *Rickettsia typhi*, accompanied by some compatible clinical and laboratory findings, including mild hypoalbuminemia (nadir 2.9 g/dL), borderline hyponatremia (nadir 135 mEq/L) and mildly elevated liver enzymes (Tsioutis et al., 2017). A major limitation of this report is the absence of convalescent serologic testing, as a definitive diagnosis of *R. Typhi* commonly requires demonstration of at least a fourfold rise in IgM and IgG titers between acute and convalescent samples (Dhawan et al., 2020; Miller et al., 2024). The patient was empirically treated with doxycycline, with convalescent serologies planned at outpatient follow-up with his infectious disease physician but was ultimately lost to follow up. While co-infections of histoplasmosis with other diseases such as tuberculosis and *Mycobacterium avium* have been reported, these have occurred almost exclusively in the context of immunocompromised patients (Caceres and Valdes, 2019; Basso et al., 2020). To our knowledge, co-occurrence of histoplasmosis and positive *Rickettsia typhi* serology has not been previously reported in the literature. This finding, along with the observation of disseminated histoplasmosis in an immunocompetent patient residing outside a traditionally endemic area, highlights the importance of maintaining clinical vigilance for endemic mycoses.

## Conclusion

This case demonstrates that histoplasmosis can develop in immunocompetent individuals outside endemic regions following high-risk environmental exposure, particularly in occupational settings involving soil disruption or stagnant water. In regions where *histoplasma capsulatum* is not traditionally endemic, clinicians must maintain a high index of suspicion in patients presenting with unexplained systemic symptoms and pulmonary infiltrates especially when initial antibiotic therapy fails. A thorough occupational and exposure history, multidisciplinary collaboration, early and broad diagnostic testing, and prompt antifungal treatment are essential to reducing morbidity. This report reinforces the need for increased awareness of occupational fungal infections, even among healthy, working-age adults.

## Data Availability

Publicly available datasets were analyzed in this study. This data can be found here: Dataset obtained from the supplementary materials of this prior publication https://pmc.ncbi.nlm.nih.gov/articles/PMC10319749/.
